# Risk factors for inpatient mortality among children with severe acute malnutrition in Zimbabwe and Zambia

**DOI:** 10.1038/s41430-023-01320-9

**Published:** 2023-08-08

**Authors:** Jonathan P. Sturgeon, Wadzanai Mufukari, Joice Tome, Cherlynn Dumbura, Florence D. Majo, Deophine Ngosa, Kanta Chandwe, Chanda Kapoma, Kuda Mutasa, Kusum J. Nathoo, Claire D. Bourke, Robert Ntozini, Mutsa Bwakura-Dangarembizi, Beatrice Amadi, Paul Kelly, Andrew J. Prendergast, the HOPE-SAM study team, Jonathan P. Sturgeon

**Affiliations:** 1 Zvitambo Institute for Maternal and Child Health Research, 16 Lauchlan Avenue, Harare, Zimbabwe; 2 Tropical Gastroenterology and Nutrition Group, University of Zambia, Lusaka, Zambia; 3 Faculty of Medicine and Health Sciences, University of Zimbabwe, Harare, Zimbabwe; 4 Centre for Genomics and Child Health, Blizard Institute, Queen Mary University of London, 4 Newark Street, London, E1 2AT UK; 5 Present Address: Centre for Genomics and Child Health, Blizard Institute, Queen Mary University of London, 4 Newark Street, London, E1 2AT UK

**Keywords:** Nutrition, Paediatrics, Risk factors

## Abstract

**Background/Objectives:**

Malnutrition underlies 45% of deaths in children under-5 years annually. Children hospitalised with complicated severe acute malnutrition (SAM) have unacceptably high mortality. We aimed to identify variables from early hospital admission (baseline factors) independently associated with inpatient mortality in this cohort to identify those most at risk.

**Subjects/Methods:**

Observational study of 745 children aged 0–59 months admitted with complicated SAM at three hospitals in Zimbabwe/Zambia. Children underwent anthropometry and clinical assessment by a study physician within 72 h of enrolment, and caregivers provided sociodemographic data. Children were followed-up daily until discharge/death. A multivariable survival analysis identified the baseline factors independently associated with mortality.

**Results:**

70/745 (9.4%) children died in hospital. Age between 6–23 months [aHR 6.53, 95%CI 2.24–19.02], higher mid-upper arm circumference [aHR 0.73, 95%CI 0.59–0.89], presence of oedema [aHR 2.22, 95%CI 1.23–4.05], shock [aHR 8.18, 95%CI 3.79–17.65], sepsis [aHR 3.13, 95%CI 1.44–6.80], persistent diarrhoea [aHR 2.27, 95%CI 1.18–4.37], lack of a toilet at home [aHR 4.35, 95%CI 1.65–11.47], and recruitment at one Harare site [aHR 0.38, 95%CI 0.18–0.83] were all independently associated with inpatient mortality. Oedematous children had a significantly higher birthweight [2987 g vs 2757 g, *p* < 0.001] than those without oedema; higher birthweight was weakly associated with mortality [aHR 1.50 95%CI 0.97–2.31].

**Conclusions:**

Children with oedema, low MUAC, baseline infections, shock and lack of home sanitation had a significantly increased risk of inpatient mortality following hospitalisation for complicated SAM. Children with high-risk features may require additional care. A better understanding of the pathophysiology of SAM is needed to identify adjunctive interventions.

## Introduction

Undernutrition remains a serious global health concern, underlying 45% of deaths in children under 5 years, mostly in low-income countries [1]. This has led the United Nations to declare a decade of action on nutrition, to reduce this mortality and morbidity burden by 2025 [2]. Severe acute malnutrition (SAM) is the form of undernutrition with the highest case fatality rate. It is characterised by wasting (very low weight-for-height or low mid-upper arm circumference (MUAC)), or bilateral nutritional oedema irrespective of the degree of wasting. SAM can be managed through therapeutic feeding in the community among children without clinical complications, but those with no appetite, severe oedema, medical complications, or clinical danger signs are categorised as having complicated SAM and are managed as inpatients. Complicated SAM is characterised by extensive enteropathy, immune dysfunction, inflammation and infections, with dysregulated metabolic pathways and extensive organ dysfunction [3, 4]. In these sickest patients, the World Health Organization (WHO) guidelines for nutritional rehabilitation are followed [5], which are based on recommendations first introduced in 1999 [6]. Despite SPHERE standards aiming for mortality of <10% as a minimum in a humanitarian setting [7], the observed inpatient mortality remains between 10–40% in many settings in sub-Saharan Africa [8]. Our recent systematic review demonstrated an average inpatient mortality of 15.7% among children hospitalised with SAM across 19 studies across eight different sub-Saharan African countries published post-2000 [9].

The causes of death among children hospitalised with SAM are dominated by symptomatic infections such as septicaemia, lower respiratory tract infection, and diarrhoea [10]. However, limited diagnostics in many settings with a high burden of SAM mean the cause of death is often not ascertained, and asymptomatic pathogen carriage is common among both adequately-nourished and undernourished children in low-income, high-pathogen environments [11]. The presence of diarrhoea significantly increases the risk of mortality [12]. Diarrhoea may be caused by enteropathogens such as shigella, cholera, or rotavirus, or occur secondary to lactose intolerance precipitated by the enteropathy-infection cycle [13, 14]. Children admitted with malnutrition have significantly more positive blood cultures than children without malnutrition [15, 16], and a higher number of pathogens initially labelled as ‘contaminants’, suggesting that immune compromise and microbial translocation secondary to malnutrition could increase susceptibility even to commensal organisms [17]. Pneumonia was associated with 2-fold higher inpatient mortality in our recent meta-analysis [9]. HIV is a significant risk factor for death in multiple studies; in addition to increasing susceptibility to infection through immunosuppression, children with concurrent HIV and SAM may have exaggerated inflammatory and metabolic perturbations [18]. Children with SAM who die are more likely to have deranged electrolytes [19], which could reflect the severity of sepsis, or be a consequence of severe diarrhoea. Finally, those admitted with malnutrition often have co-morbidities including HIV and cerebral palsy, which may contribute both to the malnourished state and the cause of death [15].

Despite the high worldwide mortality burden, studies reporting the factors associated with inpatient mortality in children with SAM remain limited: of the 19 studies since 2000 which were included in our meta-analysis, the mean number of children was 360; over three-quarters were single-centre studies and under half (8/19) were prospective studies [9]. Understanding the risk factors for mortality in different settings may help to define the population of children at highest risk, and provide insights into the mechanisms underlying inpatient mortality. Such inpatient risk factors may have longer-term implications for post-discharge mortality, morbidity and convalescent care in the community for children who survive hospitalisation [20]. Here, we report the variables from early hospital admission (‘baseline factors’) associated with inpatient mortality in a large, prospective, multi-centre study of children admitted to hospital with complicated SAM in Zimbabwe and Zambia.

## Materials and methods

### Study design

This study utilises the inpatient data from the Health Outcomes, Pathogenesis and Epidemiology of Severe Acute Malnutrition (HOPE-SAM) study. This observational cohort study, conducted between July 2016 and March 2019, recruited children aged 0–59 months hospitalised with complicated SAM in Harare Central Hospital and Parirenyatwa Hospital in Harare, Zimbabwe, and University Teaching Hospital (UTH) in Lusaka, Zambia. The full study design has been described elsewhere [4]. The protocol, standard operating procedures, and case report forms are available at https://osf.io/29uaw/.

### Participants

Eligible participants were children aged 0–59mo, admitted to one of the study hospitals with WHO SAM criteria: weight-for-height Z score (WHZ) ≤−3 using WHO growth standards, mid-upper arm circumference (MUAC) < 115 mm (for children aged >6mo), and/or bilateral pedal oedema [5]. Caregivers who did not provide written informed consent or did not wish to know their child’s HIV status, and children with known malignancy, were ineligible. Children were only enrolled during their first hospitalisation if they were admitted more than once in the study period.

### Baseline data

Participants had a comprehensive baseline assessment administered by a member of the clinical study team including a caregiver questionnaire to capture data on medical history, family history, and sociodemographic variables; weight, length/height, MUAC and body composition measurement using standardised methods; age-appropriate HIV testing; and full blood count measurement. Baseline clinical data were collected by a study doctor during examination of the child following enrolment. If the child was enrolled at the weekend, the first assessment by the study physician could be up to 72 h after enrolment; children with a baseline clinical examination after 72 h were excluded from this analysis. A brief summary is included in Supplementary Tables 1–3. The full list of available variables is shown in Form 4 [baseline health data], Form 5 [sociodemographic data], and Form 7 [clinical review by doctor], available at https://osf.io/29uaw/.

### Clinical management

Children were treated according to WHO guidelines [5]. Clinical management was conducted by ward teams, but children were reviewed daily by the study physician to collect data and to advise on management. At UTH in Zambia, children were admitted to a single closed unit for children with SAM, with dedicated staff and unit protocols. At Harare Central and Parirenyatwa Hospitals, Zimbabwe, children were admitted to the nutrition bay of several paediatric wards, and managed by shared teams with no single protocol, although country guidelines are based on the WHO 10-step approach.

### Study outcome

The primary outcome for this analysis was all-cause mortality during hospitalisation (i.e. between study enrolment and discharge from hospital); children who died after admission but prior to study enrolment were not included in this study. Children were followed up with daily study physician assessments, until the day of discharge, death, or withdrawal from the study. Participants who withdrew from the study after enrolment were censored at time of withdrawal.

### Statistical Analysis

We first selected a set of baseline variables based on their plausible association with inpatient mortality (Supplementary Fig. 1). Variables with >10% missing data, or fewer than 10 children in each category of the variable, were excluded. Differences between sites in each variable were tested using ANOVA. Individual variables were first univariably tested for association with mortality by Cox’s proportional hazards model. Age categories of <6mo, 6–23mo and 24–59mo were used, consistent with other studies [19, 21–23]. Selection of variables for inclusion in the final Cox proportional hazards model used backwards stepwise elimination, with an exit value of *p* > 0.10. Significance for independent association with inpatient mortality was set at *p* < 0.05.

Validation of the selection of variables in the final Cox proportional hazards model was conducted using bootstrap replicates, with the number of replicates set at 10 times the number of observations in the study. Each bootstrap replicate underwent backwards stepwise elimination in the Cox proportional hazards model with an exit value of *P* > 0.10. Variables which appeared in 50% of the bootstrap samples were carried forward to the final model, consistent with cut-offs used in other studies using bootstrapping for validating variable selection [24, 25]. Performance at this level has been shown to be comparable to backwards elimination [26]. The bootstrap model was then compared with the backwards elimination model for validation, ensuring both models produced concordant results. A sensitivity analysis was conducted by imputing data using multiple imputation for baseline variables with a missingness of <10%, and assessing any changes in the inference of the results.

Analyses were carried out using STATA (StataCorp. 2021. Stata Statistical Software: Release 17. College Station, TX: StataCorp LLC).

### Sample size

The sample size calculation for HOPE-SAM was based on recruiting up to 800 children, assuming 15% mortality and 15% loss to follow-up, which would provide >80% power to detect absolute differences of 17% in binary outcomes or 0.33 standard deviations for continuous outcomes between HIV-positive children with SAM and HIV-negative children with SAM, assuming an HIV prevalence of 40% [4].

## Results

**Fig. 1:**
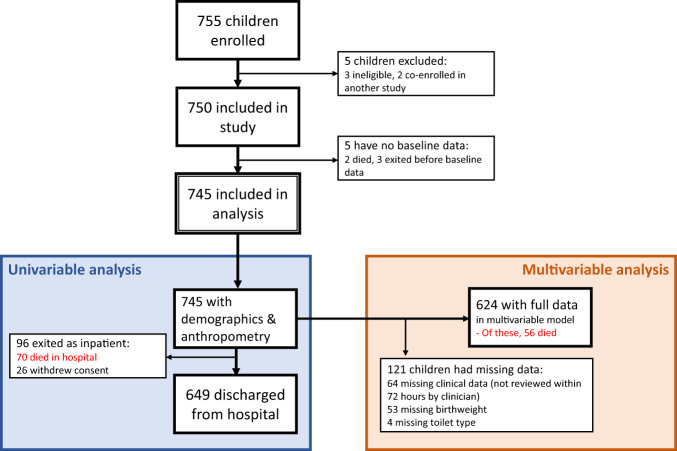
Enrolment in HOPE-SAM study. Flow diagram of children recruited in the HOPE-SAM study.

The enrolment flow is shown in Fig. 1. Of 745 children enrolled, 70 (9.4%) died in hospital, and 26 (3.5%) withdrew from the study and their data were censored at time of withdrawal; 649 children were discharged from hospital. 681/745 children (91.4%) had full baseline clinical data available from a study physician assessment within 72 h of enrolment. The children who withdrew did not have significantly different age, MUAC, WHZ score, HIV status, oedema, or sex (data not shown).

The baseline characteristics of study participants are shown in Table 1. During the study period, mortality was higher at UTH than at Harare Central or Parirenyatwa Hospitals, with half (54%) of all participant deaths occurring there. Children admitted to the Zambian site had significantly lower WHZ and mid-upper-arm circumference, were less likely to have oedematous SAM, were more likely to have HIV, and spent longer in hospital until discharge or death, compared with children admitted to the Zimbabwean sites.

**Table 1:** Baseline characteristics.

	Zimbabwe		Zambia	
Baseline data	Harare Central Hospital	Parirenyatwa	UTH	*p*-value^a^
*n*	277	226	242	
Inpatient mortality, *n*	9 (3.2%)	23 (10.2%)	38 (15.7%)	<0.001
Participant withdrew, *n*	8 (2.9%)	2 (0.9%)	16 (6.6%)	0.06
Time until death/discharge, days; mean ± SD	8.3 ± 8.6	9.3 ± 9.7	13.1 ± 7.9	<0.001
Age, mo ± SD	19.3 ± 8.8	18.4 ± 9.4	17.8 ± 7.7	0.92
Male, *n* (%)	155 (55.6%)	103 (45.6%)	132 (54.5%)	0.012
*Nutritional status*				
Length, mean (cm) ± SD	73.0 ± 2.9	72.2 ± 8.0	71.9 ± 6.1	0.22
Weight, mean (kg) ± SD	7.31 ± 1.94	6.96 ± 1.92	6.76 ± 1.45	0.22
WHZ score, mean ± SD	−2.67 ± 2.00	−2.88 ± 1.75	−3.21 ± 1.64	<0.001
MUAC, mean (cm) ± SD	12.2 ± 16.5	11.9 ± 16.4	11.3 ± 15.4	<0.001
HAZ score, mean ± SD	−3.03 ± 1.68	−2.91 ± 1.49	−3.06 ± 1.47	0.53
Oedematous	193 (69.7%)	145 (64.2%)	142 (58.7%)	0.032
Birthweight (kg), mean ± SD	2.90 ± 0.60	2.88 ± 0.58	2.92 ± 0.64	0.75
Premature cessation of breastfeeding (<12 months)	72/276 (26.1%)	57/226 (25.2%)	64/237 (27.0%)	0.83
*Clinical diagnoses*				
HIV positive	47 (17.0%)	40 (18.0%)	75 (30.1%)	<0.001
- On ART	17/47 (36.1%)	19/40 (47.5%)	30/75 (40.0%)	0.76
Maternal HIV	84/264 (31.8%)	68/213 (31.9%)	113/233 (48.5%)	0.83
HIV-exposed uninfected child	42/235 (17.9%)	33/193 (17.1%)	49/193 (25.4%)	0.13
Pneumonia^b^	35/251 (13.9%)	34/201 (16.9%)	22/229 (9.6%)	0.17
Urinary tract infection^b,c^	1/251 (0.4%)	3/201 (1.5%)	0/229 (0%)	0.33
Dermatosis^b^	50/251 (20.0%)	33/201 (16.4%)	48/229 (21.0%)	0.71
Sepsis^b^	15/251 (6.0%)	15/201 (7.5%)	17/229 (7.4%)	0.52
Meningitis^b,c^	0/251 (0%)	1/201 (0.5%)	0/229 (0%)	N/A
*Clinical signs*^b^				
Shock	12/251 (4.8%)	12/201 (6.0%)	6/229 (2.6%)	0.26
Hypothermia	19/251 (7.6%)	24/201 (11.9%)	4/229 (1.8%)	0.015
Hypoglycaemia^c^	2/251 (0.8%)	4/201 (2.0%)	0/229 (0%)	<0.001
Fever	129/251 (51.4%)	105/201 (52.2%)	90/229 (39.3%)	0.010
Diarrhoea: acute	105/251 (41.8%)	90/201 (44.8%)	67/229 (29.5%)	0.001
Diarrhoea: persistent	40/251 (15.9%)	25/201 (12.4%)	33/229 (14.4%)	0.15
Dehydration	51/251 (20.3%)	45/201 (22.4%)	40/229 (17.5%)	0.36
Cough	121/251 (48.1%)	102/201 (50.7%)	70/229 (30.6%)	0.001
Oral thrush	66/251 (26.3%)	42/201 (20.9%)	19/229 (8.6%)	<0.001
Lack of appetite	151/251 (60.2%)	104/200 (52.0%)	143/228 (62.7%)	0.61
Seizures^c^	4/251 (1.6%)	4/201 (2.0%)	1/227 (0.4%)	0.46
*Home environment*^d^				
Residence: Urban	210/274 (76.6%)	159/226 (70.4%)	82/239 (34.3%)	<0.001
Toilet availability: None	15/275 (5.5%)	10/226 (4.4%)	5/237 (2.1%)	0.005
Toilet availability: unimproved^e^	3/275 (1.1%)	3/226 (1.3%)	51/237 (21.5%)	<0.001
Toilet availability: improved	257/275 (93.5%)	213/226 (94.2%)	181/237 (76.4%)	0.005
Number of other children <5y in household, ± SD	1.51 ± 0.72	1.44 ± 0.66	1.56 ± 0.97	0.46

^a^Differences between sites tested using ANOVA test.

^b^Conditions were recorded by the study physician following history, examination of the child, and review of diagnostic tests undertaken as part of their routine clinical care.

^c^Due to fewer than 10 cases per variable, hypoglycaemia (*n* = 6), meningitis (*n* = 1), seizures (*n* = 9), and urinary tract infection (*n* = 4) were not carried forward to the multivariable analysis.

^d^Reported by caregiver questionnaire.

^e^Unimproved toilets include: Flush/pour flush to elsewhere (not into a pit, septic tank, or sewer), pit, latrine without slab, bucket latrines, hanging toilet/latrine, no facilities/bush/field/flying toilets (open defecation).

### Univariable results

Univariable analysis is shown in Table 2. Children aged 6–23mo were significantly more likely to die as an inpatient than those aged 0–6mo or 24–59mo [HR 2.93, 95%CI 1.18–7.29], *p* = 0.021]. Children admitted to Harare Central Hospital in Zimbabwe had a lower inpatient mortality compared with other sites [HR 0.34, 95%CI 0.16–0.79]. A higher MUAC was strongly associated with reduced inpatient mortality, with a 24% reduction in mortality per 1 cm increase in MUAC [HR 0.76, 95%CI 0.65–0.87, *p* < 0.001]. Similarly, each one unit increase in WHZ was associated with a 20% decreased risk of death [HR 0.80, 95%CI 0.71–0.90, *p* < 0.001]. Of the clinical signs, symptoms and diagnoses recorded by the study physician at baseline, eight were associated with mortality: shock [HR 7.68, 95%CI 4.06–14.51, *p* < 0.001], acute diarrhoea [HR 1.97, 95%CI 1.10–3.52, *p* = 0.02], persistent diarrhoea [HR 2.86, 95%CI 1.44–5.72], lack of appetite [HR 2.07, 95%CI 1.15–3.72. *p* = 0.015], pneumonia [HR 2.02, 95%CI 1.15–3.54, *p* = 0.014], hypothermia [HR 2.40, 95%CI 1.18–4.89, *p* = 0.016], respiratory distress [HR 1.88, 95%CI 1.08–3.25, *p* = 0.025], and sepsis [HR 3.34, 95%CI 1.83–6.10, *p* < 0.001]. Maternal HIV [HR 1.57, 95%CI 0.97–2.56, *p* = 0.069] and not having access to any type of toilet in the household [HR 2.21, 95%CI 0.91–4.92, *p* = 0.081] both showed weak evidence of association with child mortality. In univariable analysis, presence of oedema was not significantly associated with increased mortality. There was weak evidence that children with HIV had a higher inpatient mortality than those without HIV [HR 1.58, 95%CI 0.98–2.62, *p* = 0.06].

**Table 2:** Univariable and multivariable associations between baseline factors and in-patient mortality.

	Died in Hospital		Univariable Unadjusted Hazard Ratio	*p*-value	Multivariable Adjusted Hazard ratio (aHR) following backwards elimination	*p*-value
	Yes	No				
**Age: (*n*)**						
<6mo	1	19	0.75 [0.09–6.49]	0.80		
6–23mo	64	525	2.93 [1.18–7.29]	0.021	**6.53 [2.24–19.02]**	**0.001**
24–59mo	5	131	1			
*Continuous: age, mean; mo* *(included for information only)*	15.3	18.9	0.96 [0.93–0.99]	0.017		
**Sex: (*n*)**						
Male	35	355	1			
Female	35	320	1.17 [0.73–1.87]	0.52		
**Site: (*n*)**						
Zimbabwe: Harare Central	9	268	0.34 [0.16–0.79]	0.003	**0.38 [0.18–0.83]**	**0.014**
Zimbabwe: Parirenyatwa	23	203	0.90 [0.53–1.53]	0.70		
Zambia: UTH	38	204	1			
**Time in Hospital, days; mean (SD)**	9.99 ± 14.9	10.1 ± 8.1	N/A^a^			
**Anthropometry**						
WHZ, mean (SD)	−4.01 ± 1.69	−2.79 ± 1.80	0.80 [0.71–0.90]	<0.001		
MUAC, cm; mean (SD)	10.6 ± 1.3	11.9 ± 1.7	0.76 [0.65–0.87]	<0.001	**0.73 [0.59–0.89]**	**0.002**
HAZ, mean (SD)	−3.15 ± 1.68	−2.98 ± 1.55	1.03 [0.89–1.20]	0.66		
**Oedema**: Yes	44	436	1.27 [0.78–2.09]	0.34	**2.22 [1.23–4.05]**	**0.009**
No	26	236	1			
**Signs/Symptoms:**						
**Presence of Shock**: *Yes*	12	18	7.68 [4.06–14.51]	<0.001	**8.18 [3.79–17.65]**	**<0.001**
*No*	49	602	1			
**Hypothermia**: *Yes*	9	38	2.40 [1.18–4.89]	0.016	2.13 [0.91–5.01]	0.082
*No*	52	582	1			
**Hypoglycaemia**: *Yes*	3	3	0.99 [0.97–1.01]	0.51		
*No*	56	574	1			
**Fever**: *Yes*	32	292	1.25 [0.76–2.07]	0.38		
*No*	29	328	1			
**Diarrhoea**: *None*	20	301	1			
*Acute*	27	235	1.97 [1.10–3.52]	0.02		
*Persistent*	14	84	2.86 [1.44–5.72]	0.003	**2.27 [1.18–4.37]**	**0.014**
**Dehydration**: *Yes*	24	37	1.01 [0.95–1.07]	0.71		
*No*	37	507	1			
**Cough**: *Yes*	30	263	1.10 [0.67–1.83]	0.70		
*No*	31	357	1			
**Oral Thrush**: *Yes*	47	505	0.99 [0.87–1.13]	0.90		
*No*	14	113	1			
**Lack of Appetite**: *Yes*	46	352	2.07 [1.15–3.72]	0.015		
*No*	15	266	1			
**Respiratory distress**: *Yes*	20	86	1.88 [1.08–3.25]	0.025		
*No*	41	534	1			
**Abnormal Heat Rate**: *Yes*	7	55	1.22 [0.55–2.74]	0.62		
*No*	42	474	1			
**Seizures**: *Yes*	1	8	1.01 [0.99–1.03]	0.32		
*No*	59	609	1			
**Diagnosis**						
**Pneumonia**: *Yes*	18	73	2.02 [1.15–3.54]	0.014	1.78 [0.93–3.44]	0.083
*No*	43	547				
**UTI**: *Yes*	1	3	0.96 [0.66–1.39]	0.83		
*No*	60	614	1			
**Dermatosis**: *Yes*	18	113	0.99 [0.91–1.09]	0.91		
*No*	43	505	1			
**Sepsis**: *Yes*	14	33	3.34 [1.83–6.10]	<0.001	**3.13 [1.44–6.80]**	**0.001**
*No*	47	587	1			
**Meningitis**: *Yes*	0	1	N/A			
*No*	61	618				
***TB****: Yes*	19	127	0.97 [0.79–1.20]	0.79		
*No*	42	491				
**Medical History:**						
**HIV**: *Yes*	27	135	1.60 [0.98–2.62]	0.06		
*No*	43	540				
*(of these): On ART: Yes*	19	47	3.70 [1.62–8.46]	0.002		
*No*	8	88	1			
**Premature**: *Yes*	9	91	0.94 [0.62–1.41]	0.75		
*No*	59	569	1			
**Previous SAM Admission**: *Yes*	10	97	0.72 [0.35–1.46]	0.36		
*No*	55	563	1			
**Cerebral Palsy**: *Yes*	2	30	0.51 [0.13–2.10]	0.35		
*No*	59	589				
**Child HIV exposed, uninfected**: *Yes*	13	111	1.12 [0.61–2.05]	0.71		
*No*	57	564				
**Birthweight (kg, mean):**	2.96 ± 0.60	2.90 ± 0.61	1.22 [0.81–1.83]	0.34	1.50 [0.97–2.31]	0.065
**Family History**						
**Maternal HIV**: *Yes*	37	228	1.57 [0.97–2.56]	0.069		
*No*	31	414	1			
# of children in home, mean	1.41	1.54	0.81 [0.57–1.15]	0.25		
**Prem. Cessation br’feeding**: *Yes*	20	173	1.15 [0.68–1.94]	0.60		
*No*	48	498	1			
**Residence**: *Urban*	37	414	1			
*Peri-urban*	22	153	0.67 [0.33–1.33]	0.26		
*Rural*	10	103	0.70 [0.30–1.63]	0.41		
**Toilet**: *None*	6	24	2.12 [0.91–4.92]	0.081	**4.35 [1.65–11.47]**	**0.003**
*Unimproved*	5	52	0.75 [0.30–1.88]	0.54		
*Improved*	58	593	1			

Results show the hazard ratio, and the 95% confidence interval, along with the associated *p* value. Results bolded in multivariable analysis have an adjusted hazard ratio (aHR) with a *p* < 0.05. All aHR values with a *p* < 0.10 are included in the final model. Variables not meeting the *p* < 0.10 cut-off during stepwise elimination are not included in the multivariable model.

^a^not available (N/A) as the time is used for the proportional hazard ratio.

### Multivariable analysis

**Fig. 2:**
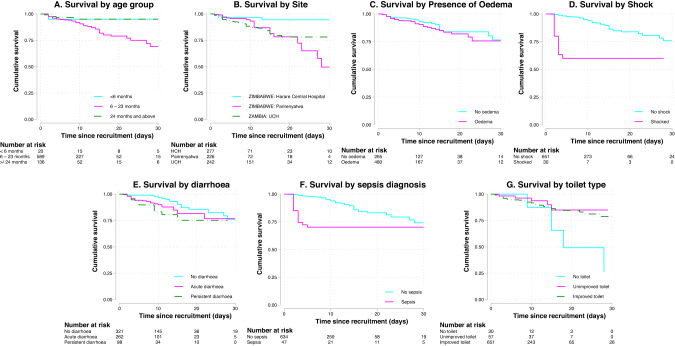
Kaplan-Meier curves for each of the seven significant categorical variables. Hazard tables are included beneath each graph. Graphs showing survival by **A** age group, **B** site, **C** presence of oedema, **D** Shock, **E** Diarrhoea, **F** Sepsis and **G** access to a toilet.

The backwards elimination model included 11 baseline variables in the final Cox proportional hazards model, of which 8 were significantly independently associated with mortality (Fig. 2). Children aged 6–23 months, compared with older children (24–59 months), had 6-fold higher mortality [aHR 6.53, 95%CI 2.24–19.02, *p* = 0.001]. Children managed at Harare Central Hospital, compared with other sites, had 62% lower mortality [aHR 0.38, 95%CI 0.18–0.83; *p* = 0.014]. Children with oedematous, compared to non-oedematous, SAM had 2-fold higher mortality [aHR 2.22, 95%CI 1.23–4.05, *p* = 0.009]. Every 1 cm rise in MUAC was associated with a 27% reduction in mortality [aHR 0.73, 95%CI 0.59–0.89, *p* = 0.002]. Three clinical factors were associated with increased mortality: presence of shock [aHR 8.18, 95%CI 3.79–17.65, *p* < 0.001], persistent diarrhoea [aHR 2.27, 95%CI 1.18–4.37, *p* = 0.014], and sepsis [aHR 2.13, 95%CI 1.44–6.80, *p* = 0.001]. Finally, having no toilet at home was associated with 4-fold increased mortality, compared to children from households with an improved toilet [aHR 4.35, 95%CI 1.65–11.47, *p* = 0.003].

Hypothermia, pneumonia, and birthweight were retained in the final model (*p* < 0.10) but had weak evidence of association with mortality (Table 2). The validation approach using bootstrap sampling identified the same 11 variables for inclusion in the final model, and the same 8 factors were independently associated with inpatient mortality (Supplementary Table 5). The final model included 624 children with full clinical data collected within 72 hours of enrolment (Fig. 1/Supplementary Table 4). A sensitivity analysis using multiple imputation for missing variables did not change the inference of the findings (data not shown).

## Discussion

We identified eight characteristics present during early hospital admission that were independently associated with subsequent inpatient mortality among children admitted with complicated SAM to three hospital sites in Zimbabwe and Zambia. Presence of oedema and low MUAC were both associated with increased mortality, and children aged 6–23mo had higher mortality than younger and older children. Three baseline clinical presentations were associated with mortality: shock, persistent diarrhoea, and sepsis. Mortality differed between sites in this study, and children living in households without access to an improved toilet had higher mortality than those with an improved toilet at home. Collectively, these findings from a large, observational, multi-centre, prospective study in two southern African countries identify children with the highest risk of dying in hospital, who may require additional monitoring and/or adjunctive interventions to reduce their risk of mortality.

Age and nutritional status were associated with mortality. Children aged 6–23 months were significantly more likely to die than older or younger children. This may partly reflect differences in the pathophysiology of SAM between ages. Children under six months, who are predominantly breastfed, may have different underlying causative factors including prematurity, intra-uterine growth restriction or congenital anomalies [27]. However, due to the small numbers in the under-six-month group (*n* = 20, of whom one died), it was not possible to stratify analyses to explore whether risk factors for mortality differed at this age. Young infants also are not classified with SAM based on MUAC, so classification of SAM below 6 months may identify a different group of children compared to other ages. Older children (24–59mo) may have differences in physiological reserve, body composition or aetiology compared to younger children. Higher MUAC was associated with lower inpatient mortality, with each 1 cm increase associated with a 27% mortality reduction. The selection of MUAC over WHZ following backwards elimination in the final model reflects the strong co-linearity between these two variables (Pearson correlation coefficient = 0.67, *p* < 0.01), and may represent the fact that MUAC is more sensitive than WHZ at detecting SAM in the 6–23 month age group [28], while WHZ can be difficult to interpret in children with oedema. Other studies which found WHZ to be independently predictive of mortality did not include MUAC [29, 30]. In the absence of MUAC, WHZ would have remained a significant independent predictor of inpatient mortality in our study [aHR 0.83, 95%CI 0.70–0.98, *p* = 0.035] (with other factors being retained).

Presence of oedema was associated with 2-fold higher inpatient mortality in multivariable but not in univariable analyses. Although a relationship between oedema and mortality is consistent with several studies [12, 31], other smaller cohorts have not identified oedema as a risk factor [19, 21, 32], and in meta-analysis oedema was not associated with mortality [9]. Oedematous SAM typically presents more acutely than non-oedematous SAM. MUAC remains a significant predictor of mortality in children with oedema, highlighting the independent contribution of sarcopenia to mortality, regardless of oedema status. This distinction was drawn in previous definitions of severe malnutrition, which included wasting (marasmus), oedema (kwashiorkor), and oedematous wasting (marasmic-kwashiorkor), with the latter group noted to have the highest mortality [33]. The mechanism underpinning oedema remains poorly understood. Some studies have suggested that distinct epigenetic programming in protein, amino acid, and/or lipid metabolism leads some children, but not others, to develop oedema in response to the same nutritional insult [34, 35]. A retrospective study from Jamaica suggested that prenatal or intrauterine factors contribute, since children with oedematous malnutrition had significantly higher birthweight than children with non-oedematous malnutrition [36]. In our cohort, children with oedema also had significantly higher birthweight than children without oedema (mean 2987 g v 2757 g, *p* < 0.001), even after adjusting for HIV, prematurity and sex, consistent with the hypothesis that intra-uterine factors alter the subsequent physiological response to malnutrition. We found some evidence that increased birthweight was associated with higher mortality in this cohort [aHR 1.50, 95%CI 0.97–2.31, *p* = 0.065], after adjusting for oedema. Whether this reflects an increased risk of inpatient mortality in those with higher birthweight who develop SAM, earlier mortality in infants with lower birthweight prior to developing SAM, or different management strategies in these children requires further study.

Several clinical variables demonstrated a strong association with mortality. The presence of shock was associated with 8-fold increased mortality. Shock arises from a variety of causes including sepsis, dehydration, and heart failure, all of which are common in children with complicated SAM [9]. Given the multifactorial causes of complicated SAM, and the severity of the condition, it is unsurprising that shock was associated with the highest hazard of death in this cohort, similar to the studies included in our recent meta-analysis of inpatient mortality [9]. Children with shock tended to die early during hospitalisation (Fig. 2D). Presence of persistent diarrhoea could represent chronic infection or enteropathy, and causes loss of nutrients, dehydration, and a vicious cycle of enteropathy, impaired mucosal healing, and malnutrition [37]. Although persistent diarrhoea is classified as three or more loose or watery stools a day for at least 14 days, our baseline data relied on caregiver recall. Diarrhoea frequently complicates SAM, and independently predicted mortality in our meta-analysis of six studies of children with SAM [9]. For this study we distinguished persistent diarrhoea from acute diarrhoea, and only persistent diarrhoea significantly independently predicted mortality.

Since children with SAM frequently die of symptomatic infections [38], it was not unexpected that sepsis independently predicted inpatient mortality. There was weaker evidence for pneumonia predicting mortality. It has been noted that the causative organisms of sepsis and pneumonia in malnourished children may differ from well-nourished children [39, 40]. This situation is compounded by the lack of microbiology laboratory facilities to identify organisms and resistance patterns in many clinical settings. Further study is needed on alternative first-line antibiotic regimens, and when to switch empirically to second-line antibiotics.

Not having access to a toilet was significantly independently associated with mortality. Whilst this may represent an increased mortality risk directly associated with lack of sanitation, several recent WASH trials found no reductions in diarrhoea following installation of improved sanitation [41, 42], and this variable may be a proxy for more complex socioeconomic factors associated with poverty. Since this factor is not a clinical characteristic per se, further studies of this association could be valuable to inform preventative household-level interventions for complicated SAM and the associated mortality risk when these children require hospital admission.

In children who survived to discharge and for whom vital status was known one-year post-discharge (*n* = 608, 93.7% of those discharged), our previous analyses showed that *lack* of oedema [aHR 2.23, 95%CI 1.24–4.01], presence of HIV [aHR 3.83, 95%CI 2.15–6.82], ongoing low WHZ score/low MUAC [aHR 2.28, 95%CI 1.22–4.25], and cerebral palsy [aHR 5.60, 95%CI 2.72–11.50] were all independently associated with post-discharge mortality [20]. This contrasts with the current inpatient results, especially with regard to presence of oedema, and may suggest that different processes underlie longer-term mortality: those with oedema tend to die earlier, as an inpatient, whilst sarcopenia and long-term wasting are associated with later mortality.

Finally, there were significant differences in risk of inpatient mortality between hospital sites. The inpatient mortality at UTH, Zambia was 5-fold higher than at Harare Central Hospital, Zimbabwe. This may partly reflect the different demographics among children admitted to UTH, including a lower WHZ score, lower MUAC, higher HIV prevalence, and higher rural or peri-urban residence, compared with those admitted to Zimbabwean sites. The length of stay was longer in UTH at 13 days, compared with 8–9 days for the sites in Zimbabwe (*p* < 0.01), which increased the time at risk for inpatient mortality, and may reflect more severely ill children presenting in Zambia. It is notable that outpatient mortality was somewhat higher for the children admitted to Zimbabwean sites, suggesting that earlier discharge may simply alter the timing of death [20], but other factors such as seasonality, may also be reflected.

This is the largest prospective study examining independent factors associated with inpatient mortality among children with complicated SAM since WHO guidelines were introduced in 2000, and the second largest overall, including older studies [9]. The study had full data available for 624/745 (83.8%) participants, all of whom were included in the final analysis; however, we lacked full data for 14 of the 70 (20%) deaths due to early mortality (Supplementary Table 4). Some prior studies had full data on fewer than half of participants, reducing their power significantly [30]; a key consideration for a condition which is characterised by clinical heterogeneity and high case fatality. Limitations of our study include delays in recruiting children after hospitalisation, due to the time taken for caregivers to consider enrolment, which means that early deaths (<24 h) were typically not included, and the study population is therefore not reflective of all children with SAM presenting to hospital. The mortality in the HOPE-SAM cohort (9.4%) was just below the SPHERE standard for humanitarian settings of 10% [7], but above the WHO standard of 5% [5], and likely underestimated overall mortality because the highest-risk children died prior to enrolment in the study. Additionally, there was a lack of diagnostic testing to identify pertinent additional risk factors. Although being the largest prospective study, some variable categories still had small numbers of children. Finally, to select variables for the multivariable model we used backwards stepwise regression. Although we did validate it with a bootstrapping approach, this stepwise method has been criticised for potentially allowing some true explanatory variables to drop out, and allowing nuisance variables (those statistically significant but unrelated to the underlying processes) to be selected in [43].

Children hospitalised with complicated SAM continue to have unacceptably high inpatient mortality [9]. The variables independently predicting mortality in this study highlight the multifactorial nature of the disease, and those children most at risk of death following admission with SAM. Further work is required to gain a better understanding of the underlying pathophysiology of SAM to target interventions to children at highest risk.

### Supplementary information

Supplemental Material

## Data Availability

The data can be made from the corresponding author on reasonable request.

## References

[1] Black RE, Victora CG, Walker SP, Bhutta ZA, Christian P, de Onis M (2013). Maternal and child undernutrition and overweight in low-income and middle-income countries. Lancet.

[2] World Health Organization, Food and Agriculture Organization of the United Nations. Driving commitment for nutrition within the UN Decade of Action on Nutrition: policy brief. Geneva: World Health Organization; 2018 (WHO/NMH/NHD/17.11).

[3] Thaxton GE, Melby PC, Manary MJ, Preidis GA (2018). New insights into the pathogenesis and treatment of malnutrition. Gastroenterol Clin.

[4] Bwakura-Dangarembizi M, Amadi B, Bourke CD, Robertson RC, Mwapenya B, Chandwe K (2019). Health Outcomes, Pathogenesis and Epidemiology of Severe Acute Malnutrition (HOPE-SAM): rationale and methods of a longitudinal observational study. BMJ Open.

[5] World Health Organization. Guideline: updates on the management of severe acute malnutrition in infants and children. Geneva: World Health Organization; 2013. 24649519

[6] World Health Organization. Management of severe malnutrition: a manual for physicians and other senior health workers. Geneva: World Health Organization; 1999.

[7] Thurstans S, Turnbull P, Velly D, Middleton W (2011). 2011 Edition of the Sphere Handbook Humanitarian Charter and Minimum Standards in Humanitarian Response. Field Exch.

[8] Tickell KD, Denno DM (2016). Inpatient management of children with severe acute malnutrition: a review of WHO guidelines. Bull World Health Organ.

[9] Karunaratne R, Sturgeon JP, Patel R, Prendergast AJ (2020). Predictors of inpatient mortality among children hospitalized for severe acute malnutrition: a systematic review and meta-analysis. Am J Clin Nutr.

[10] Ndlovu S, David-Govender C, Tinarwo P, Naidoo KL (2022). Changing mortality amongst hospitalised children with Severe Acute Malnutrition in KwaZulu-Natal, South Africa, 2009–2018. BMC Nutr.

[11] Amadi B, Zyambo K, Chandwe K, Besa E, Mulenga C, Mwakamui S (2021). Adaptation of the small intestine to microbial enteropathogens in Zambian children with stunting. Nat Microbiol.

[12] Irena AH, Mwambazi M, Mulenga V (2011). Diarrhea is a major killer of children with severe acute malnutrition admitted to inpatient set-up in Lusaka, Zambia. Nutr J.

[13] Fagundes-Neto U, de Andrade JA (1999). Acute diarrhea and malnutrition: lethality risk in hospitalized infants. J Am Coll Nutr.

[14] Nyeko R, Kalyesubula I, Mworozi E, Bachou H (2010). Lactose intolerance among severely malnourished children with diarrhoea admitted to the nutrition unit, Mulago hospital, Uganda. BMC Pediatr.

[15] Harris C, Mills R, Seager E, Blackstock S, Hiwa T, Pumphrey J (2019). Paediatric deaths in a tertiary government hospital setting, Malawi. Paediatr Int Child Health.

[16] Talbert A, Thuo N, Karisa J, Chesaro C, Ohuma E, Ignas J (2012). Diarrhoea complicating severe acute malnutrition in Kenyan children: a prospective descriptive study of risk factors and outcome. PLoS One.

[17] Abrha A, Abdissa A, Beyene G, Getahun G, Girma T (2011). Bacteraemia among severely malnourished children in jimma university hospital, ethiopia. Ethiop J Health Sci.

[18] Heikens GT, Bunn J, Amadi B, Manary M, Chhagan M, Berkley JA (2008). Case management of HIV-infected severely malnourished children: challenges in the area of highest prevalence. Lancet.

[19] Rytter MJ, Babirekere-Iriso E, Namusoke H, Christensen VB, Michaelsen KF, Ritz C (2016). Risk factors for death in children during inpatient treatment of severe acute malnutrition: a prospective cohort study. Am J Clin Nutr.

[20] Bwakura-Dangarembizi M, Dumbura C, Amadi B, Ngosa D, Majo FD, Nathoo KJ (2021). Risk factors for postdischarge mortality following hospitalization for severe acute malnutrition in Zimbabwe and Zambia. Am J Clin Nutr.

[21] Bachou H, Tumwine JK, Mwadime RKN, Tylleskär T (2006). Risk factors in hospital deaths in severely malnourished children in Kampala, Uganda. BMC Pediatr.

[22] Girum T, Kote M, Tariku B, Bekele H (2017). Survival status and predictors of mortality among severely acute malnourished children <5 years of age admitted to stabilization centers in Gedeo Zone: a retrospective cohort study. Ther Clin Risk Manag.

[23] Desyibelew HD, Baraki AG, Dadi AF (2019). Mortality rate and predictors of time to death in children with severe acute malnutrition treated in Felege-Hiwot Referral Hospital Bahir Dar, Northwest Ethiopia. BMC Res Notes.

[24] Bunea F, She Y, Ombao H, Gongvatana A, Devlin K, Cohen R (2011). Penalized least squares regression methods and applications to neuroimaging. NeuroImage.

[25] Blackstone EH (2001). Breaking down barriers: helpful breakthrough statistical methods you need to understand better. J Thorac Cardiovasc Surg.

[26] Austin PC (2008). Bootstrap model selection had similar performance for selecting authentic and noise variables compared to backward variable elimination: a simulation study. J Clin Epidemiol.

[27] Vygen SB, Roberfroid D, Captier V, Kolsteren P (2013). Treatment of severe acute malnutrition in infants aged <6 months in Niger. J Pediatr.

[28] Tessema M, Laillou A, Tefera A, Teklu Y, Berger J, Wieringa FT (2020). Routinely MUAC screening for severe acute malnutrition should consider the gender and age group bias in the Ethiopian non-emergency context. PLoS One.

[29] Chimhuya S, Kambarami RA, Mujuru H (2007). The levels of malnutrition and risk factors for mortality at Harare Central Hospital-Zimbabwe: an observation study. Cent Afr J Med.

[30] Oumer A, Mesfin F, Demena M (2016). Survival Status and Predictors of Mortality among Children Aged 0-59 Months Admitted with Severe Acute Malnutrition in Dilchora Referral Hospital, Eastern Ethiopia. East Afr J Health Biomed Sci.

[31] Nhampossa T, Sigaúque B, Machevo S, Macete E, Alonso P, Bassat Q (2013). Severe malnutrition among children under the age of 5 years admitted to a rural district hospital in southern Mozambique. Public Health Nutr.

[32] Nabukeera-Barungi N, Grenov B, Lanyero B, Namusoke H, Mupere E, Christensen VB (2018). Predictors of mortality among hospitalized children with severe acute malnutrition: a prospective study from Uganda. Pediatr Res.

[33] Amadi B, Kelly P, Mwiya M, Mulwazi E, Sianongo S, Changwe F (2001). Intestinal and systemic infection, HIV, and mortality in Zambian children with persistent diarrhea and malnutrition. J Pediatr Gastroenterol Nutr.

[34] Jahoor F, Badaloo A, Reid M, Forrester T (2005). Protein kinetic differences between children with edematous and nonedematous severe childhood undernutrition in the fed and postabsorptive states. Am J Clin Nutr.

[35] May T, de la Haye B, Nord G, Klatt K, Stephenson K, Adams S, et al. One-carbon metabolism in children with marasmus and kwashiorkor. EBioMedicine. 2022;75. 10.1016/j.ebiom.2021.103791. 10.1016/j.ebiom.2021.103791 PMC8761690 35030356

[36] Forrester TE, Badaloo AV, Boyne MS, Osmond C, Thompson D, Green C (2012). Prenatal factors contribute to the emergence of kwashiorkor or marasmus in severe undernutrition: evidence for the predictive adaptation model. PLoS One.

[37] Prendergast AJ, Kelly P (2016). Interactions between intestinal pathogens, enteropathy and malnutrition in developing countries. Curr Opin Infect Dis.

[38] Bourke CD, Berkley JA, Prendergast AJ (2016). Immune Dysfunction as a Cause and Consequence of Malnutrition. Trends Immunol.

[39] Ginsburg AS, Izadnegahdar R, Berkley JA, Walson JL, Rollins N, Klugman KP (2015). Undernutrition and pneumonia mortality. Lancet Glob Health.

[40] Berkley JA, Lowe BS, Mwangi I, Williams T, Bauni E, Mwarumba S (2005). Bacteremia among children admitted to a rural hospital in Kenya. N. Engl J Med.

[41] Humphrey JH, Mbuya MNN, Ntozini R, Moulton LH, Stoltzfus RJ, Tavengwa NV (2019). Independent and combined effects of improved water, sanitation, and hygiene, and improved complementary feeding, on child stunting and anaemia in rural Zimbabwe: a cluster-randomised trial. Lancet Glob Health.

[42] Null C, Stewart CP, Pickering AJ, Dentz HN, Arnold BF, Arnold CD (2018). Effects of water quality, sanitation, handwashing, and nutritional interventions on diarrhoea and child growth in rural Kenya: a cluster-randomised controlled trial. Lancet Glob Health.

[43] Smith G (2018). Step away from stepwise. J Big Data.

