# Supplementary material for: Risk factors for inpatient mortality among children with severe acute malnutrition in Zimbabwe and Zambia
Source: Eur J Clin Nutr. Author manuscript; Available in PMC 2023 Sep 4. (PMC10473959; doi:10.1038/s41430-023-01320-9)
Supplement: Supplementary Material [file EMS184993-supplement-Supplementary_Material.docx]

**Risk factors for inpatient mortality among children with severe acute malnutrition in Zimbabwe and Zambia**

**HOPE-SAM study team**

Beatrice Amadi^3^, Ellen Besa^3^, Claire D Bourke^2^, Mutsa Bwakura-Dangarembizi^1,4^, Bernard Chasekwa^1^, Pamela Chakara^1^, Leah Chidamba^1^, Theodore Chidawanyika^1^, Tafadzwa Chidhanguro^1^, Kapula Chifunda^3^, Esther Chilala^3^, Lovemore Chingaoma^1^, Miyoba Chipunza^3^, Nivea Chulu^3^, Adlight Dandadzi^1^, Tenzeni Dumba^3^, Cherlynn Dumbura^1^, Washington Dune^1^, Temwaninge Gondwe^3^, Margaret Govha^1^, Karen Gwanzura^1^, Jean H Humphrey^1,5^, Tomola Kaingu^3^, Chanda Kapoma^3^, Sarudzai Kasaru^3^, Lydia Kazhila^3^, Paul Kelly^2,3^, Lucy Macwani^3^, Florence D Majo^1^, Idah Mapurisa^1^, Patience Mashayanembwa^1^, Faithful Masimba^1^, Stephen Moyo^1^, Eddington Mpofu^1^, Mary Mpundu^3^, Edith Mukusho^3^, Johnson Mushonga^1^, Kuda Mutasa^1^, Agatha Muyenga^3^, Sophreen Mwaba^3^, Simutanyi Mwakamui^3^, Mpala Mwanza^3^, Benjamin Mwapenya^1^, Kusum J Nathoo^4^, Gwendolyn Nayame^3^, Deophine Ngosa^3^, Sibongile Nkiwane^1^, Robert Ntozini^1^, Penias Nyamwino^1^, Evelyn Nyendwa^3^, Dennis Phiri^3^, Phillipa Rambanepasi^1^, Andrew J Prendergast^1,2^, Ruairi Robertson^2^, Sandra Rukobo^1^, Thompson Runodamoto^1^, Virginia Sauramba^1^, Shepherd Seremwe^1^, Pururudzai Simango^1^, Jonathan P Sturgeon^1,2^, Jonathan R Swann^6^, Andreck Tembo^3^, Dalitso Tembo^3^, Blessing Tsenesa^1^, Jonathan CK Wells^7^, Kanekwa Zyambo^3^, and Khozya Zyambo^3^.

^1^Zvitambo Institute for Maternal and Child Health Research, 16 Lauchlan Avenue, Harare, Zimbabwe.

^2^Centre for Genomics and Child Health, Blizard Institute, Queen Mary University of London, 4 Newark Street London, E1 2AT, UK

^3^Tropical Gastroenterology and Nutrition Group, University of Zambia, Lusaka, Zambia

^4^Faculty of Medicine and Health Sciences, University of Zimbabwe, Harare, Zimbabwe

^5^Department of International Health, Johns Hopkins Bloomberg School of Public Health, 615 North Wolfe Street, Baltimore, MD 21205, USA

^6^Faculty of Medicine, Department of Metabolism, Digestion and Reproduction, Imperial College, London, UK

^7^Population, Policy & Practice Dept. UCL GOS Institute of Child Health, University College London, UK

**Supplemental Figure 1:** Conceptual framework of signs, symptoms, and systems plausibly associated with mortality


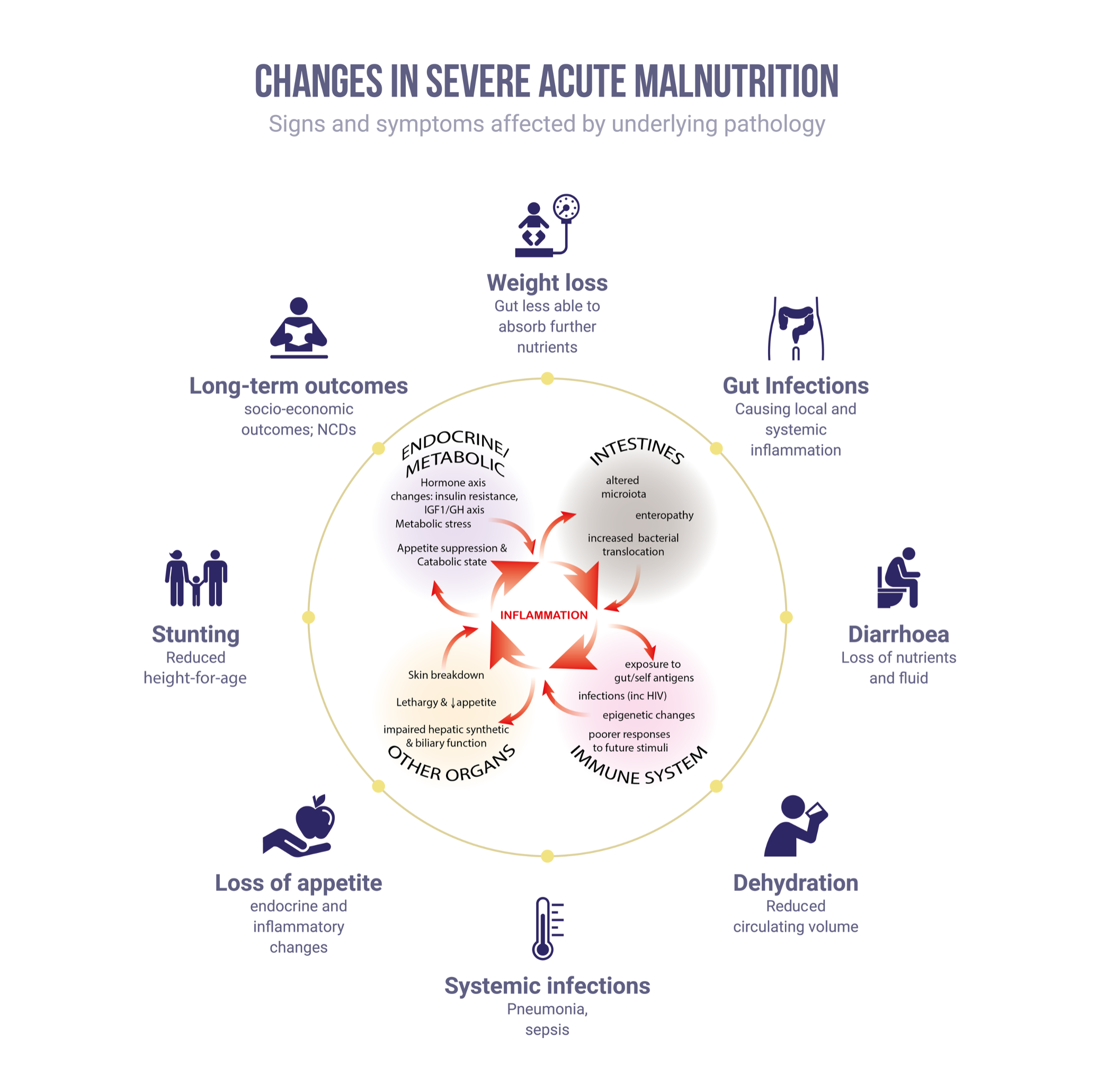


|  | **Form 4: Admission Details** |
| --- | --- |
|  | **Full form: https://osf.io/29uaw/files/osfstorage/5b55f22fc86a8c0012458241** |
| **Identification** | Participant ID (PID) |
|  | Sex |
|  | Date of birth |
|  | Age |
|  | Date of initial assessment in hospital |
|  | Time of initial assessment in hospital |
|  | Are you the main person who cares for the child each day? |
|  | What is your relationship to the child? |
|  | Where is the mother? |
|  | How long has your child been unwell with this current illness? |
|  | Who did you seek help from prior to hospitalization? |
|  | Has the child already been admitted to hospital during this current episode of illness? |
| **Current History** | **In the last 2 weeks:** |
|  | Fever/hot body? |
|  | Cold hands and feet? |
|  | Night sweats? |
|  | Cough? |
|  | Fast or difficult breathing? |
|  | Diarrhoea? (3 or more loose or watery stools in 24 hours) |
|  | If yes, how many days of diarrhoea has your child had in a row? |
|  | How long ago did this diarrhoea problem first begin? |
|  | **Last 24h? Or in the last 2 weeks?** |
|  | Blood in the stools? |
|  | Vomiting? |
|  | Poor or reduced appetite? |
|  | Weight loss (or poor weight gain)? |
|  | Swelling of any part of their body? |
|  | Convulsions / fits? |
|  | Drowsy or difficult to wake? |
|  | Has your child experienced any other symptoms during this illness? |
|  | Other problem? |
| **Medication** | Has your child taken any medications or nutritional supplements in the past two weeks? |
|  | Has he/she taken any antibiotics in the past 2 weeks? [+which] |
|  | Has he/she taken any TB medications in the past 2 weeks? [+which] |
|  | Has he/she taken any antiretroviral (anti-HIV) medicines in the past 2 weeks? [which] |
|  | Has he/she taken any nutritional supplements in the past 2 weeks? |
|  | Has he/she taken any other medication in the past 2 weeks? |
| **Past medical History** | What sort of delivery did this child have? |
|  | Was this child born premature (early)? |
|  | What was the weight of this child at birth? |
|  | What was the length of this child at birth? |
|  | Has your child had any of these medical conditions now or in the past? |
|  | Is this child currently being breastfed? |
|  | How old was the child when he/she stopped breastfeeding? |
|  | Why did you stop breastfeeding? |
|  | Is this child currently receiving solid foods? |
|  | At what age did he/she first have solid foods? |
|  | Has this child ever been admitted to hospital and received therapeutic milk (e.g. F75) before this episode? |
|  | How many times has your child been admitted to hospital and received therapeutic milk (not including this episode) |
|  | Has this child ever been treated for malnutrition in the community? |
|  | How many episodes of malnutrition have been treated in the community (not including this episode)? |
|  | Has your child ever had persistent diarrhoea (loose stools for at least 14 days in a row)? |
| **HIV history** | What was your last HIV test result? |
|  | When was that test? |
|  | Was the mother HIV positive during pregnancy, delivery or breastfeeding of this child? |
|  | Is the mother currently taking ARV's? |
|  | When were these ARVs started? |
|  | What is the HIV status of the child? |
|  | Was the child diagnosed with HIV prior to this hospital admission? |
|  | Is the child currently taking cotrimoxazole? |
|  | Is the child currently taking ARV's? |
|  | When were these ARV's started? |
|  | Which drugs is the child taking? |
| **Anthropometry** | Weight |
|  | Length |
|  | MUAC |
|  | Head circumference |
|  | Oedema |

**Supplemental Table 1: Summary of the questions asked in the admission case report form, Form 4**

This is a brief summary. For the full questions, including the coding for each question please see the full form available from: <https://osf.io/29uaw/files/osfstorage/5b55f22fc86a8c0012458241>

|  | **Form 5 (Baseline)** |
| --- | --- |
|  | **Full form: https://osf.io/29uaw/files/osfstorage/5b55f22c69e43a0010d458b9** |
| Family | Is the child’s mother alive? |
|  | Is the child’s father alive? |
|  | How many people live in the child’s homestead/household? |
|  | How many of these are children below 5 years of age? |
|  | What is your marital status? |
|  | How old are you? |
|  | What is your highest completed grade of education? |
|  | How do you earn a living? |
|  | What is your husband/partner’s highest completed grade of education? |
|  | How does your husband/partner earn a living? |
|  | What is the total income of your household per month? |
|  | Where do you live the majority of the time? |
|  | Is there a toilet or latrine of any type at the homestead/household? |
|  | What kind of toilet/latrine is it? |
|  | How many households share this toilet facility? |
|  | Where do you get water from? |
|  | Do you treat your drinking water? |
|  | Where do you cook? |
|  | What methods do you use to cook? |
|  | Does your household have any electricity from the mains (e.g. ZESA/ZESCO? ) |
|  | Do you receive any food aid from the government or other organizations? |
|  | What food aid items have you received in the past 12 months? |

**Supplemental Table 2: Summary of the questions asked in the admission case report form, Form 5**

This is a brief summary. For the full questions, including the coding for each question please see the full form available from: <https://osf.io/29uaw/files/osfstorage/5b55f22c69e43a0010d458b9>

|  | **Form 7 (clinical)** |
| --- | --- |
|  | **Full form: https://osf.io/29uaw/files/osfstorage/5b55f235c86a8c00114543a6** |
|  | Full problem list |
|  | Includes dehydration, shock, hypothermia, fever, poor appetite, hypoglycaemia, eye signs, heart failure, oedema, diarrhoea, vomiting, respiratory distress, cough, sepsis, meningitis, measles in past 3 months, malaria, ear discharge, HIV infected, HIV exposed, TB, dermatosis, skin infection, oral thrush, cannula site infection, UTI, abnormal electrolytes, cerebral palsy, seizures, other |
|  | Observations |
|  | Examination - including general, adjuncts, cardiovascular, respiratory, gastroenterology, neurology and other |
| Resuscitation | What signs of shock did the child have in the past 24 hours? |
|  | Did the child receive any intravenous fluids for shock in the past 24 hours? |
|  | What intravenous fluids did the child receive for shock? |
|  | What was the total volume of intravenous infusions given in past 24 hours? |
|  | Did the child receive a blood transfusion for anaemia/pallor in the past 24 hours? |
|  | What type of blood transfusion? |
|  | What was the total volume of blood given in the past 24 hours? |
|  | What oral/nasogastric fluids did the child receive in the past 24 hours (apart from F75/F100 feeds) |
|  | Did the child receive any ACTIVE rewarming in the past 24 hours? |
|  | Medications |
|  | Clinical Tests |

**Supplemental Table 3: Summary of the questions asked in the clinical case report form, Form 7**

This is a brief summary. For the full questions, including the coding for each question please see the full form available from: <https://osf.io/29uaw/files/osfstorage/5b55f235c86a8c00114543a6>

| Missing Variables | Freq. | Percent of total | Cumulative total | Number of deaths in this group |
| --- | --- | --- | --- | --- |
| Anthropometry | 0 | 0% | 0% | - |
| Clinical only | 59 | 7.92% | 7.92% | 7/59 |
| Birthweight only | 51 | 6.85% | 14.77% | 5/51 |
| Toilet type only | 4 | 0.54% | 15.31% | 0/4 |
| Birthweight and toilet type | 2 | 0.27% | 15.58% | 0/2 |
| Clinical and birthweight | 4 | 0.54% | 16.12% | 1/4 |
| Clinical, birthweight, and toilet type | 1 | 0.13% | 16.25% | 1/1 |
| None [all present] | **624** | **83.76%** | **100%** | **56/675** |
| Total | **745** | **100** |  | **70/745** |
|  |  |  |  |  |
| Variables Removed before multivariable analysis due to missingness | | | |  |
| Electrolytes | **540** | **80.32%** |  |  |
| Heart rate | **167** | **22.42%** |  |  |

**Supplementary Table 4**: Missingness of the data for the multivariable model

| Variable | Obs | Mean % inclusion | Std. Dev |
| --- | --- | --- | --- |
| Age category | **7,450** | **93%** | **0.251394** |
| Gender | 7,450 | 27% | 0.444579 |
| Site | **7,450** | **70%** | **0.459393** |
| WHZ | 7,450 | 35% | 0.475868 |
| MUAC | **7,450** | **60%** | **0.490418** |
| HAZ | 7,450 | 23% | 0.422359 |
| Oedema | **7,450** | **61%** | **0.486652** |
| Shock | **7,450** | **99%** | **0.099835** |
| Hypothermia | **7,450** | **56%** | **0.496814** |
| Fever | 7,450 | 19% | 0.391102 |
| Diarrhoea | **7,450** | **71%** | **0.454811** |
| Cough | 7,450 | 27% | 0.445882 |
| Oral thrush | 7,450 | 35% | 0.478515 |
| Poor appetite | 7,450 | 35% | 0.476128 |
| Respiratory Distress | 7,450 | 28% | 0.448766 |
| Pneumonia | **7,450** | **57%** | **0.494869** |
| Dermatosis | 7,450 | 33% | 0.47194 |
| Sepsis | **7,450** | **75%** | **0.433158** |
| TB | 7,450 | 37% | 0.482819 |
| HIV | 7,450 | 19% | 0.395008 |
| preterm | 7,450 | 33% | 0.470703 |
| Prior SAM admission | 7,450 | 31% | 0.463265 |
| Cerebral Palsy | 7,450 | 9% | 0.2884 |
| HEU | 7,450 | 26% | 0.438517 |
| Birth weight | **7,450** | **52%** | **0.499715** |
| Premature cessation of breastfeeding | 7,450 | 19% | 0.395424 |
| Residence | 7,450 | 19% | 0.38884 |
| Toilet type | **7,450** | **76%** | **0.425962** |
| More than 1 other child >5y | 7,450 | 23% | 0.423037 |
| Maternal HIV status | 7,450 | 36% | 0.481302 |

**Supplementary Table 5**: Results of the bootstrap validation of the variable selection for the final multivariable model
